# Positron emission tomography-computed tomography and 4-hydroxynonenal-histidine immunohistochemistry reveal differential onset of lipid peroxidation in primary lung cancer and in pulmonary metastasis of remote malignancies

**DOI:** 10.1016/j.redox.2017.01.005

**Published:** 2017-01-11

**Authors:** Nevenka Piskač Živković, Mladen Petrovečki, Čedna Tomasović Lončarić, Igor Nikolić, Georg Waeg, Morana Jaganjac, Kamelija Žarković, Neven Žarković

**Affiliations:** a Clinical Hospital Dubrava, Zagreb, Croatia; b Karl Franzen's University of Graz, Institute of Molecular Biosciences, Austria; c Toxicology and Multipurpose Dept., Anti-Doping Lab, Doha, Qatar; d University of Zagreb School of Medicine, Department of Pathology, Clinical Hospital Center, Zagreb, Croatia; e Rudjer Boskovic Institute, LabOS, Zagreb, Croatia

**Keywords:** PET-CT, Positron Emission Tomography-Computed Tomography, 4-HNE, 4-hydroxynonenal, 18F-FDG, 18-fluorodeoxyglucose, SPN, solitary pulmonary nodule, LDHA, Lactate dehydrogenase A, SUV, standardized uptake value, ROS, reactive oxygen species, RNS, reactive nitrogen species, RALBP1, Ral-binding protein1, Nrf2, nuclear factor erythroid 2-related factor, Keap1, Kelch-like ECH-associated protein 1, TAM, tumor-associated macrophages, VEGF, vascular endothelial growth factor, NSCLC, non-small cell lung cancer, VATS, video-assisted thoracoscopic surgery, GSH, glutathione, mAb, monoclonal antibodies, hydroxyl radical OH^-^, superoxide O_2_^-^, N, nitrogen oxide, NO_2_, nitrogen dioxide, H_2_O_2_, hydrogen peroxide, HOCl, hypochloric acid, O_3_, ozone, NADPH, nicotinamide adenine dinucleotide phosphate Nox oxidase, iNOS, inducible nitric oxide synthase, MMP, matrix metalloproteinases, Lipid peroxidation (LPO), 4-hydroxynonenal (HNE), Positron emission tomography and computed tomography (PET-CT), Cell growth regulation, Inflammation, Immunohistochemistry

## Abstract

The Aim of the study was to reveal if PET-CT analysis of primary and of secondary lung cancer could be related to the onset of lipid peroxidation in cancer and in surrounding non-malignant lung tissue.

**Methods:**

Nineteen patients with primary lung cancer and seventeen patients with pulmonary metastasis were involved in the study. Their lungs were analyzed by PET-CT scanning before radical surgical removal of the cancer. Specific immunohistochemistry for the major bioactive marker of lipid peroxidation, 4-hydroxynonenal (HNE), was done for the malignant and surrounding non-malignant lung tissue using genuine monoclonal antibody specific for the HNE-histidine adducts.

**Results:**

Both the intensity of the PET-CT analysis and the HNE-immunohistochemistry were in correlation with the size of the tumors analyzed, while primary lung carcinomas were larger than the metastatic tumors. The intensity of the HNE-immunohistochemistry in the surrounding lung tissue was more pronounced in the metastatic than in the primary tumors, but it was negatively correlated with the cancer volume determined by PET-CT. The appearance of HNE was more pronounced in non-malignant surrounding tissue than in cancer or stromal cells, both in case of primary and metastatic tumors.

**Conclusions:**

Both PET-CT and HNE-immunohistochemistry reflect the size of the malignant tissue. However, lipid peroxidation of non-malignant lung tissue in the vicinity of cancer is more pronounced in metastatic than in primary malignancies and might represent the mechanism of defense against cancer, as was recently revealed also in case of human liver cancer.

## 1 Introduction

**Fig. 1:**
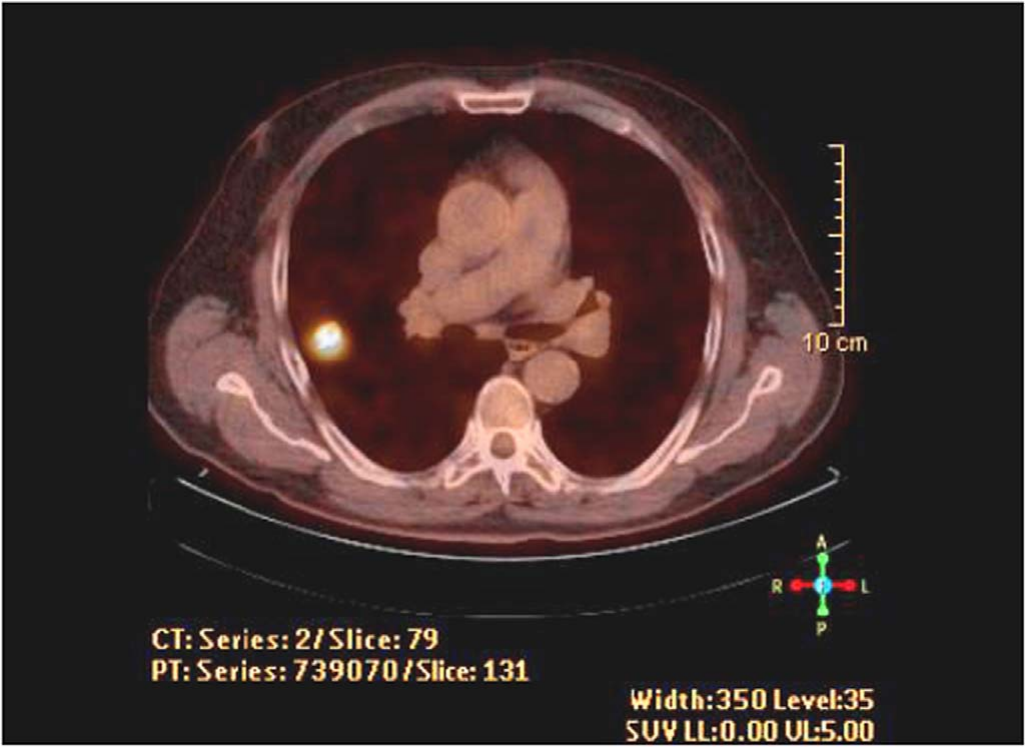
PET-CT scan of solitary malignant pulmonary nodule reflecting cancer growth as shining spot (SUVmax 5.0).

The basic feature of a tumor cell is the metabolic switch from oxidative phosphorylation to anaerobic glycolysis even under circumstances of normal oxygen saturation, a phenomenon known as the Warburg effect [1]. Lactate dehydrogenase (LDHA) catalyzes the conversion of pyruvate to lactate, and is considered the key enzyme that regulates anaerobic metabolism, including the increased expression of the Glut-1 to Glut-4 transport proteins [2], [3]. The mentioned tumor cell feature is used in the diagnostic method of Positron Emission Tomography and Computed Tomography (PET-CT) scanning. Instead of glucose, the glucose analog 18-fluorodeoxyglucose (18F-FDG) is used. Cancer cells phosphorylate 18F-FDG by hexokinase to FDG-6-phosphate, a compound which is not further metabolized but accumulates in the cell. The intensity of 18F-FDG accumulation is quantitatively expressed by standardized uptake value (SUVmax) [4], [5], [6]. The metabolic activity of the tumor expressed as SUVmax is in correlation with the intensity of anaerobic glycolysis of malignant cells and the number of metabolically active cancer cells. In the majority of clinical studies, a SUV of >2.5 is considered the cut-off value indicating a malignant etiology of the pulmonary nodule [7], [8], [9], [10], [11], [12] (Fig. 1).

Oxidative stress is defined as an imbalance in the cellular redox reactivity towards oxidation, resulting in an increased production of reactive oxygen species (ROS) and reactive nitrogen species (RNS). Reactive oxygen and nitrogen species include various substances like the oxygen free radicals superoxide (O_2_^-^) and hydroxyl radical (OH^-^), the nitrogen free radicals nitrogen oxide (NO) and nitrogen dioxide (NO_2_), as well as molecules such as hydrogen peroxide (H_2_O_2_), hypochloric acid (HOCl) and ozone (O_3_). Among ROS the most reactive are free radicals, which contain an unpaired electron in their outer shells [13], [14]. ROS may lead to irreversible damage in macromolecules such as DNA, proteins and lipids, where the occurrence of self-catalyzed chain reaction of lipid peroxidation plays a particular role, with the subsequent production of reactive aldehydes out of which 4-hydroxynonenal (HNE) is of major interest due to its numerous biological effects. Thus, HNE is known also as “second messenger of free radicals", as it lives longer than free radicals and can migrate within the cell gaining biological activities that resemble free radicals. Additionally, HNE is able to bind to macromolecules and thereby modify their function even if not being toxic [15], [16]. The effects of HNE are ranging from cell growth regulation, intracellular signal transmission and communication with the cellular environment eventually leading to cytotoxicity and cell death, which mainly depend on concentrations of HNE, the character of the biomolecules involved and the type of cells affected by HNE [17], [18], [19], [20], [21]. In various malignant cells ROS levels are permanently elevated due to the malignant cell transformation and increased metabolism, whereas their antioxidant levels are often decreased [22], [23], [24]. Although such unfavorable conditions should normally lead to apoptosis and cell death, instead uncontrolled tumor cell proliferation occurs, while the tumor-specific antioxidant protection system neutralizes the high ROS levels. A possible cause could be an alternative antioxidant protection route by the multispecific transport protein Ral-binding protein1 (RALBP1) and its conjugation potential with the HNE molecule [25], as well as the increased activity of transcription factor nuclear factor erythroid 2-related factor 2 (Nrf2) responsible for antioxidant synthesis within the cell. Increased Nrf2 activity has been noted in malignant lung tumor cells, and is in correlation with chemoresistance and radioresistance of tumors. Increased Nrf2 activity in malignant cells is the result of a mutation in the cytoplasmic molecule Keap1 (Kelch-like ECH-associated protein 1), which in conjunction with Nrf2 causes its inactivation [26], [27], [28]. Hence, the multipotent role of the HNE molecule is demonstrated by its potential to create a HNE-Keap1 complex [29], as well as the HNE-His type of protein adducts, thereby directly affecting the tumor antioxidant protection system, but also causing inflammation [30].

The association between chronic inflammation and tumors has been proven repeatedly in numerous experimental and epidemiological studies. An important feature of tumors is their potential for chemotaxis and inflammatory cell activation [31]. The role of tumor-associated macrophages (TAM) has been thoroughly researched. The activated macrophages show an increased expression of the enzyme group nicotinamide adenine dinucleotide phosphate (NADPH) oxidase (Nox) and inducible nitric oxide synthase (iNOS). They stimulate tumor invasion by releasing the matrix metalloproteinases MMP-2 and MMP-9, which break down the extracellular matrix and basal membrane [32]. TAMs also stimulate tumor angiogenesis by releasing vascular endothelial growth factor (VEGF) and endothelin-2 urokinase-type plasminogen activator [33], [34]. The host-to-tumor relationship can therefore be manifested by increased accumulation of inflammatory cells in the vicinity of the tumor and the release of ROS and second messengers of oxidative stress, such as HNE molecules, which have the potential to affect the signaling pathways in cancer cells [35], [36].

By comparing the metabolic activity of the tumor in different PET/CT scans with the immunohistochemistry findings of HNE in the malignant cells, tumor stroma and in normal tissue adjacent to the tumor, the aim of this research was to determine whether the tumor affects the surrounding tissue by the spread of oxidative stress or whether the surrounding tissue aims to affect the tumor as a reflection of the host reaction, by way of an inflammatory reaction and HNE generated within malignant tissue and in the surrounding non-malignant tissue.

## 2 Patients, materials and methods

### 2.1 Patients

Nineteen patients (52.8%) with primary lung tumors and 17 patients (47.2%) with secondary lung tumors were involved in this study.

In the group of patients with primary lung tumors majority suffered from adenocarcinoma, followed by planocellular carcinoma, non-small cell lung cancer (NSCLC), and carcinoid, while one patient was diagnosed with mesothelioma. Two out of three patients were current or former smokers, while every third patient received platinum-based chemotherapy prior to lung resection (Table 1).

In the group with secondary lung tumors, 13 patients had been pathohistologically diagnosed with metastatic adenocarcinoma of the rectosigmoid colon. The other diagnoses included metastatic breast, kidney, and laryngeal cancer. Every third patient was current or former smoker, while most of them had undergone chemotherapy prior to lung resection. The mean time from the last chemotherapy cycle to resection was 18.8 months (Table 2).

The tissue samples for pathohistological analysis were obtained after lung lobectomy or segmentectomy performed at the Department of Thoracic Surgery of the Clinical Hospital Dubrava in Zagreb. The most frequently used surgical technique was video-assisted thoracoscopic surgery (VATS), and in several patients thoracotomy was necessary due to the extent of the procedure. The indication for open biopsy of the tumor growth was established by the thoracic surgeon and the attending pulmonologist. The basic requirement for enrollment into this prospective study were positive PET-CT scans of pulmonary nodules of different size and different SUVmax value. The qualitative and quantitative PET-CT results were assessed by physicians from the Clinical Hospital Center Zagreb and the Medical Center Medikol Zagreb in accordance with the standardized diagnostic protocol.

### 2.2 Pathohistological and immunohistochemical analysis

Tissue sample collection was performed by pathologists at the Clinical Hospital Dubrava, as well as the further pathohistological and immunohistochemical analysis necessary to establish the pathohistological diagnosis. Three samples were collected from each patient; one from the tumor itself, one from the tumor and the adjacent tissue, and one from the surrounding tissue up to 10 mm from the tumor. Immediately upon resection, the samples were fixed in 10% formalin, dehydrated with ethanol, and embedded into a paraffin blocks. The paraffin blocks were then cut into 5μ thin slices, deparaffinized and rehydrated. One slice from each sample was stained with hematoxylin-eosin and analyzed by the attending pathologist from the Clinical Hospital Dubrava, while another slice from each sample was additionally prepared for immunohistochemical analysis with monoclonal antibodies (mAb) for HNE-histidine conjugate applied at a dilution of 1:10 in LabOS (Rudjer Boskovic Institute, Zagreb). This genuine monoclonal antibody, specific for the HNE-histidine adducts was prepared at the University of Graz from the culture medium of the clone derived from a fusion of Sp2-Ag8 myeloma cells with B-cells of a BALBc mouse immunized by HNE-modified keyhole limpet hemocyanine. For the immunohistochemical detection of the HNE-protein adducts the immunoperoxidase technique was used with secondary rabbit-anti-mouse antibodies (EnVision kit, DAKO, Glostrup, Denmark). Finally, the sections were incubated with diaminobenzidine (DAB, Dako Glostrup, Denmark) substrate, and counterstained with haematoxylin (Kemika, Zagreb, Croatia) as described before [18]. The immunohistochemical slides prepared in this way were re-analyzed by a second pathologist at the Clinical Hospital Center Zagreb, not knowing the previous allocation of the patients to respective study groups.

This research was approved by the Ethical Committee of the Clinical Hospital Dubrava, and informed consent was obtained from each patient prior to the surgical procedure, allowing additional testing of the tissue samples for HNE monoclonal antibodies at the Rudjer Boskovic Institute, Zagreb, as described before.

The HNE immunohistochemical positivity was determined by the usual semi-quantitative method [37], and the samples were marked in the following way:

- negative sample (0), +/- very low, hardly discernible positivity (0.5), + weakly positive (1),

++ moderately positive (2), +++ very positive sample (3).

### 2.3 Statistical analysis

The obtained results were analyzed by MedCalc using the following statistical methods: Fisher test, Chi-squared test, Pearson correlation and Mann-Whitney test. For the statistical purposes the observed variables were grouped into two categories: 0, 0.5, and 1 (weakly positive - group 1) and 2 and 3 (definitely positive - group 2). Statistical significance was established at p<0.05.

## 3 Results

No statistically significant difference was established between HNE-intensity in the cytoplasm of tumor cells that were exposed to neoadjuvant chemotherapy when compared to those who were not treated by chemotherapy. The mean time from the last chemotherapy cycle to resection was 5 months. Chemotherapy also did not affect the HNE-immunopositivity in non-malignant tissue surrounding respective cancer (Table 1, Table 2).

The mean tumor volume of 2.87 cm^3^ of the secondary tumors was significantly (p<0.01) smaller than was the mean size of primary lung cancer of 17.56 cm^3^. However, comparison of intensity of metabolic tumor activity (SUV) in respect to the tumor size between primary and secondary tumors did not show statistically significant difference in their respective SUV intensities. There was also no statistically significant difference between HNE-immunohistochemical intensity in the malignant cells of primary and of the secondary lung neoplasms. The predominant value of the HNE intensity in the cytoplasm of primary and of secondary lung tumor cells was ++(2) being the most pronounced in necrotic regions.

In the tumor stroma HNE-immunohistochemical intensities were only moderate, graded either as +/-(0.5), and +(1) or even negative, while the predominant intensity was 0.5 (+/-). There was no statistically significant difference in the HNE-intensity of inflammatory cells of the tumor stroma in either primary or secondary lung tumors. Among the inflammatory cells, macrophages (TAM) were predominant, along with a very small number of neutrophils, fibroblasts and lymphocytes.

**Fig. 2:**
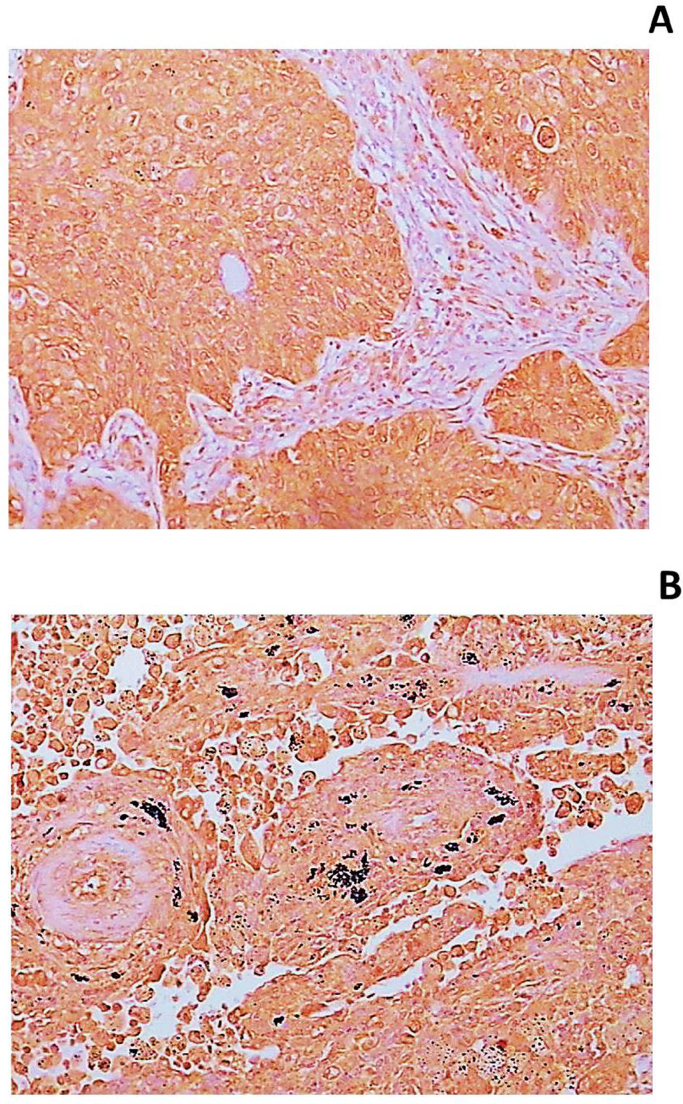
The HNE-immunohistochemical appearance of HNE-protein adducts in lung cancer. The presence of the HNE-histidine adducts is manifested by the brown-colored immunochemical staining mostly present in the cytoplasm of malignant cells, but not in the stromal cells (Fig. 2A, 400x), while in the surrounding lung tissue HNE appears diffusely present (Fig. 2B). Note the black anthracotic pigment associated with cigarette-tar deposits in the lungs on Fig. 2B.

However, a statistically significant difference was obtained when the HNE intensities of surrounding lung tissue was compared between primary and secondary lung tumors (p<0.001). In the group of primary lung tumors, a weakly positive HNE intensity of +(1) was predominant, while in the surrounding tissue of secondary lung tumors higher HNE intensities of ++(2) and +++(3) were most frequently found. No HNE-negative surroundings were recorded at all (Fig. 2).

A statistically significant difference (p=0.02) between primary and secondary lung tumors was also observed for the HNE intensities of the edematous fluid in the cancer surroundings. The inflammatory cells in the vicinity of secondary tumors also gave significantly (p<0.001) higher levels of HNE than was observed for the inflammatory cells in the vicinity of primary tumors.

Finally, the HNE-immunohistochemical intensity of the surroundings of primary and of secondary lung tumors and tumor volumes were compared, showing a statistically significant (r=−0.43, p<0.05) negative correlation between the volume of secondary lung cancer (metastasis) and the HNE-immunopositivity in surrounding lung tissue, which was not observed in case of primary lung cancer.

## 4 Discussion

The mean tumor volume of the secondary, metastatic tumors was significantly smaller than was the mean primary lung tumor volume prior to resection, probably due to the fact that the patients with metastatic cancer had been actively followed by imaging methods to monitor the onset of metastasis of their primary tumor. However, comparison of the intensity of tumor metabolic activity (SUV) and tumor size did not show statistically significant differences between the SUV intensities of primary and of secondary lung tumors. These findings confirm the known fact that at the time of establishing the diagnosis primary lung tumor cells are characterized by anaerobic glycolysis, while SUV intensity mainly depends on the number of metabolically active cells. These results are also confirmed by the fact that there was no statistically significant difference between the HNE intensities in the cytoplasm of primary and of secondary lung tumor cells.

The neoadjuvant chemotherapy used did not influence the appearance of HNE in tumors or in surrounding lungs, which is likely due to the choice of chemotherapeutic protocols as well as the period from the last cycle to lung resection (the mean time for primary tumors was 5 months, and for secondary tumors 18.8 months.

The observed correlation of the intensity of occurrence of HNE-protein conjugates in the cells with the tumor volume indicates a mutual dependence of lipid peroxidation and tumor size, which was not described before. Although one may assume that these findings suggest that lipid peroxidation enhances the tumor growth, at least because HNE is known growth factor [16], [38], we think it is actually the other way around. Namely, HNE can indeed, at least in vitro, enhance the growth of different types of cells, but only if present at very low levels that cannot be determined by immunocytochemistry or immunohistochemistry [39]. If present at higher concentrations that can be immunochemically visualized in cells and tissues, HNE exerts proapoptotic effects proportional to the level of the HNE-protein adducts [40] and can even suppress the growth of malignant stem cells by interaction with oxidatively modified extracellular matrix. Eventually, at very high concentrations, HNE can also induce tumor necrosis.

While in the initial stages of carcinogenesis malignant cells could resemble their normal counterparts and under aggressive oxidative stress manifested by increase of HNE production behave individually [41], in advanced stages the altered metabolism of cancer could lead to more uniform behavior of malignant cells. Because malignant cells have less expressed capacities of antioxidants, such as glutathione [24] and consequently use alternative, tumor-specific antioxidant mechanism to defend themselves from toxicity of oxidative stress by tumor-specific membrane associated catalase [30], which is suppressed by HNE, malignant cells are more sensitive to cytotoxicity of HNE than are non-malignant, normal cells.

Therefore, tumor progression associated with enhanced lipid peroxidation could cause the tumor decay through apoptosis or even necrosis, as was recently proposed to represent the particular mechanism of the host defense against invading cancer [30], [42]. This possibility was recently confirmed also by findings of abundant HNE present in the most necrotic regions of the lung cancer, which were also associated to systemic disturbance in lipid metabolism and generalized enhancement of the HNE production [43].

Hence, non-malignant cells in the tumor surroundings could enhance the intensity of lipid peroxidation to defend themselves from invading cancer jointly with the immuno-competent inflammatory cells. Among the inflammatory cells in the tumor stroma, macrophages (TAM) were revealed to dominate, along with a very small number of neutrophils, fibroblasts, and lymphocytes. However, there was no statistically significant difference found in HNE intensities in the inflammatory cells of the tumor stroma between primary and secondary lung tumors, while their predominant value of HNE intensity in the inflammatory cells of the tumor stroma of primary and secondary lung tumors was +/- (0.5). The occurrence of only so small amounts of oxidative stress products within tumor stroma and stromal inflammatory cells confirms the known fact that inflammatory cells in the tumor stroma are not directed against the tumor, but rather serve its function.

Opposite to that, significant difference was obtained between the HNE intensities of the surroundings of primary and of secondary lung tumors. These findings support the hypothesis that host defense against cancer may be explained by an increased activity of inflammatory cells in the surroundings of secondary lung tumors and a subsequent increased ROS/RNS synthesis. This can further lead to a locally increased permeability of the alveo-capillary membrane due to the effect on the endothelial and epithelial cells and enhanced production of HNE in non-malignant cells in the vicinity of cancer, thus causing decay of invading cancer as was shown already in the rat model of spontaneously regressing W256 carcinoma [35], [36].

Comparison of the HNE intensity of the surroundings of primary and secondary lung tumors with SUV intensity and HNE intensity of the tumor cells did not reveal statistically significant differences. Thus, HNE-mediated oxidative stress in the non-malignant tissue surrounding cancer was not caused by the tumor invasion but by the reaction of the normal cells to cancer aggression, in particular by defense generated through inflammatory process. Namely, the intensity of lipid peroxidation determined by the HNE immunopositivity of surrounding tissues was negatively correlated with the volume of secondary, but not of primary tumors, which means that there is a significant difference in the behavior of primary and secondary tumors with regard to the development of lipid peroxidation in the surrounding tissue. The occurrence of higher levels of HNE-protein conjugates in the vicinity of smaller secondary tumors could mean that HNE is generated by non-malignant cells to act as an obstacle to the growth of metastatic tumors.

In conclusion we may state that both PET-CT and HNE immunohistochemistry reflect cancer development related to the size of the malignant tissue, while non-malignant lung tissue in the vicinity of cancer expresses more pronounced lipid peroxidation in metastatic than in primary malignancies tending to defend itself from cancer, as was recently revealed in case of human liver cancer [44].

## Conflict of interest

The authors have no conflicts of interest to declare.

## Dedication

This paper is dedicated to Prof. Mladen Petrovečki who was until the last days of his life fighting against lung cancer, giving everyone the most impressive example of personal courage and supreme scientific spirit. Dear Mladen, thank you for everything, we were privileged to work with you.

## Figures and Tables

**Table 1:** Primary lung tumors.

Ordinal number	Gender	Age	Smoking	Pack/years	PHD	CH	Number of cycles	Time post CH/ months	L/Hb/Tr	HNE-T	HNE-S	Lymphangiosis	Angioinvasion	SUV	Volume cm^3^
13	F	52	YES/C	40	NSCLC	PE	4	2	3.8/119/181	2	2	–	–	6.9	4.18
26	M	67	YES/C	30	NSCLC	PE	6	4	6.8/126/371	2	1	–	–	16.3	42
45	M	63	YES/F	30	NSCLC	–	–	–	8.7/152/180	0.5	0.5	–	–	5.6	2
2	M	75	YES/F	41	carcinoid	–	–	–	8.7/152/267	2	1	–	YES	4.5	4.05
40	F	74	–	–	carcinoid	–	–	–	6.7/118/234	2	0.5	–	–	5.3	51.87
7	F	65	–	–	carcinoid	–	–	–	4.9/141/188	0.5	1	–	–	1.3	0.52
6	M	62	YES/F	34	planocellular Ca	PE	6	4	4.9/132/242	1	1	YES	–	1.8	4.18
8	M	79	YES/F	28	planocellular Ca	–	–	–	9.2/127/355	3	1	YES	YES	8.3	100
20	M	56	YES/F	42	planocellular Ca	PE	6	12	3.3/161/152	0.5	1	–	–	2.1	1.76
30	M	77	YES/F	15	planocellular Ca	–	–	–	9.1/107/232	2	2	YES	YES	12.3	56
44	M	65	–	–	planocellular Ca	PE	2	6	11.5/133/387	1	1	YES	YES	11.9	198.75
4	M	68	YES/F	80	adenocarcinoma	–	–	–	10.9/137/161	3	1	–	–	12	227.5
10	M	64	–	–	adenocarcinoma	PK	4	2	5.9/117/332	1	1	YES	YES	9.5	56
11	M	62	YES/F	20	adenocarcinoma	–	–	–	4.4/138/307	1	1	–	YES	4.7	6.8
17	M	57	YES/C	43	adenocarcinoma	–	–	–	4.6/139/322	1	1	–	YES	7.0	21
23	M	86	–	–	adenocarcinoma	–	–	–	86.4/144/155	0.5	1	–	–	10.2	14.13
26	M	67	YES/C	31	adenocarcinoma	PE	6	2	6.8/126/371	2	1	–	–	16.3	42
27	F	66	–	–	adenocarcinoma	–	–	–	12.9/99/218	2	1	YES	–	5.6	1.14
31	M	76	–	–	*meso*thelioma	–	–	–	6.5/104/237	3	1	–	–	5.3	80

Legend: YES/C - current smokers, YES/F - former smokers, CH - chemotherapy, PE - cisplatin/etoposide, PC - paclitaxel/carboplatin, HNE-T - HNE-immunohistochemical intensity in tumor cells, HNE-S - HNE-immunohistochemical intensity in non-malignant lung tissue surrounding tumor, SUV - standardized uptake value, L/Hb/Tr - leukocyte/hemoglobin g/L/platelets before resection

**Table 2:** Secondary lung tumors.

Ordinal number	Gender	Age	Smoking	Pack/years	PHD	Primary	CH	Time post CH/ months	L/Hb/Tr	HNE-T	HNE-S	SUV	Volume cm^3^
5	F	75	–	–	adenocarcinoma	rectum	bevacizumab	12	4.4/133/201	2	1	9.5	33.49
9	M	60	–	–	adenocarcinoma	rectum	5FU, bevacizumab	6	6.0/134/168	2	1	6.0	4.18
12	M	72	–	–	adenocarcinoma	sigma	5FU, bevacizumab	6	5.9/139/170	1	2	1.8	0.9
14	M	58	–	–	adenocarcinoma	rectum	5FU, bevacizumab	12	6.5/147/138	2	3	4.3	4.18
19	M	69	YES/F	34	adenocarcinoma	rectum	5FU	60	8.2/118/156	3	2	2.2	22.5
24	M	69	–	–	adenocarcinoma	rectum	5FU	24	9.7/119/156	2	3	5.3	0.11
25	M	65	YES/F	41	adenocarcinoma	rectum	5FU	6	5.0/136/156	2	2	2.2	0.26
29	M	77	YES/C	30	adenocarcinoma	rectum	5FU	6	3.4/129/170	2	1	4.3	22.5
35	M	71	–	–	adenocarcinoma	rectum	5FU	24	6.8/148/202	2	1	1.8	0.17
37	F	40	YES/C	12	adenocarcinoma	rectum	5FU	6	7.5/121/235	1	2	5.0	0.96
42	M	73	–	–	adenocarcinoma	sigmoid	5FU	6	7.2/149/246	1	2	2.7	0.69
43	M	65	–	–	adenocarcinoma	rectum	5FU	24	5.3/146/159	1	1	3.5	8.17
38	M	48	–	–	renal Ca		–	–	6.1/133/178	2	2	3.2	1.57
34	F	44	–	–	breast Ca		tamoxifen	60	8.4/124/215	1	2	11.6	0.18
39	F	50	–	–	adenocarcinoma	sigmoid	5FU	12	8.4/120/358	0.5	2	3.0	4.32
21	M	70	YES/F	28	renal Ca		–	–	7.2/144/389	2	2	4.6	4.18
36	M	74	YES/F	35	planocellular Ca	larynx	–	–	4.9/132/175	2	2	7.4	10

Legend: YES/C - current smokers, YES/F - former smokers, CH - chemotherapy, 5FU - fluorouracil, HNE-T - HNE-immunohistochemical intensity in tumor cells, HNE-S - HNE-immunohistochemical intensity in non-malignant lung tissue surrounding tumor, SUV - standardized uptake value, L/Hb/Tr - leukocyte/hemoglobin g/L/platelets before resection.
